# Supplementary material for: Spin-photon entanglement with direct photon emission in the telecom C-band
Source: Nat Commun. 2024 Nov 11;15:9740. doi: 10.1038/s41467-024-53964-1 (PMC11554671; doi:10.1038/s41467-024-53964-1)
Supplement: Supplementary file 1 — Supplementary Information [file 41467_2024_53964_MOESM1_ESM.pdf]

Supplementary information
Spin-photon entanglement with direct photon emission in the telecom C-band

P. Laccotripes *et al.*

CONTENTS

1. Sample 2

2. Experimental setup 3

3. QD state splitting and g-factor 6

4. Spin initialisation theoretical model 8

5. Ramsey interferometry - rotation pulse fidelity 10

6. Magnetic field optimisation 12

7. Quantum correlations and entanglement fidelity 14

Supplementary References 18

1. SAMPLE

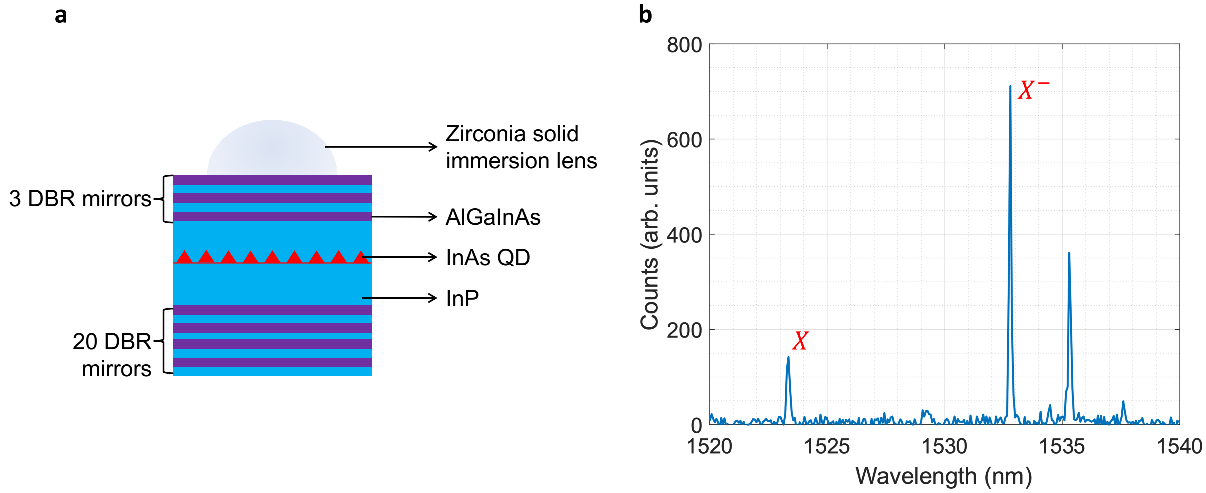

Supplementary Figure 1: **The quantum dot sample.** (a) Sample's layer structure. (b) Photoluminescence spectrum under above-band excitation. Sharp exciton, X, and negatively charged exciton, X^-, peaks are labelled.

The device used in this report was grown using Metalorganic Vapour Phase Epitaxy (MOVPE). The structure of the sample is shown in Supplementary Figure 1(a). Self-assembled Indium Arsenide (InAs) quantum dots were embedded in bulk Indium Phosphide (InP) for operation in the telecom C-band. Droplet epitaxy was used to produce symmetric dots on the (001) surface. An asymmetric Bragg cavity is also built to enhance emission efficiency from the top of the device. The DBR consists of 20 AlGaInAs/InP bottom layer pairs and 3 AlGaInAs/InP top layer pairs, where each layer has an optical thickness of $\lambda/4$. The intrinsic region consists of $3\lambda/4$ below and above the QD layer for a total cavity length of $3\lambda/2$. Finally, a zirconia solid immersion lens is added to enhance the extraction efficiency. The photoluminescence spectrum of our QD under above-band excitation is shown in Supplementary Figure 1(b).

2. EXPERIMENTAL SETUP

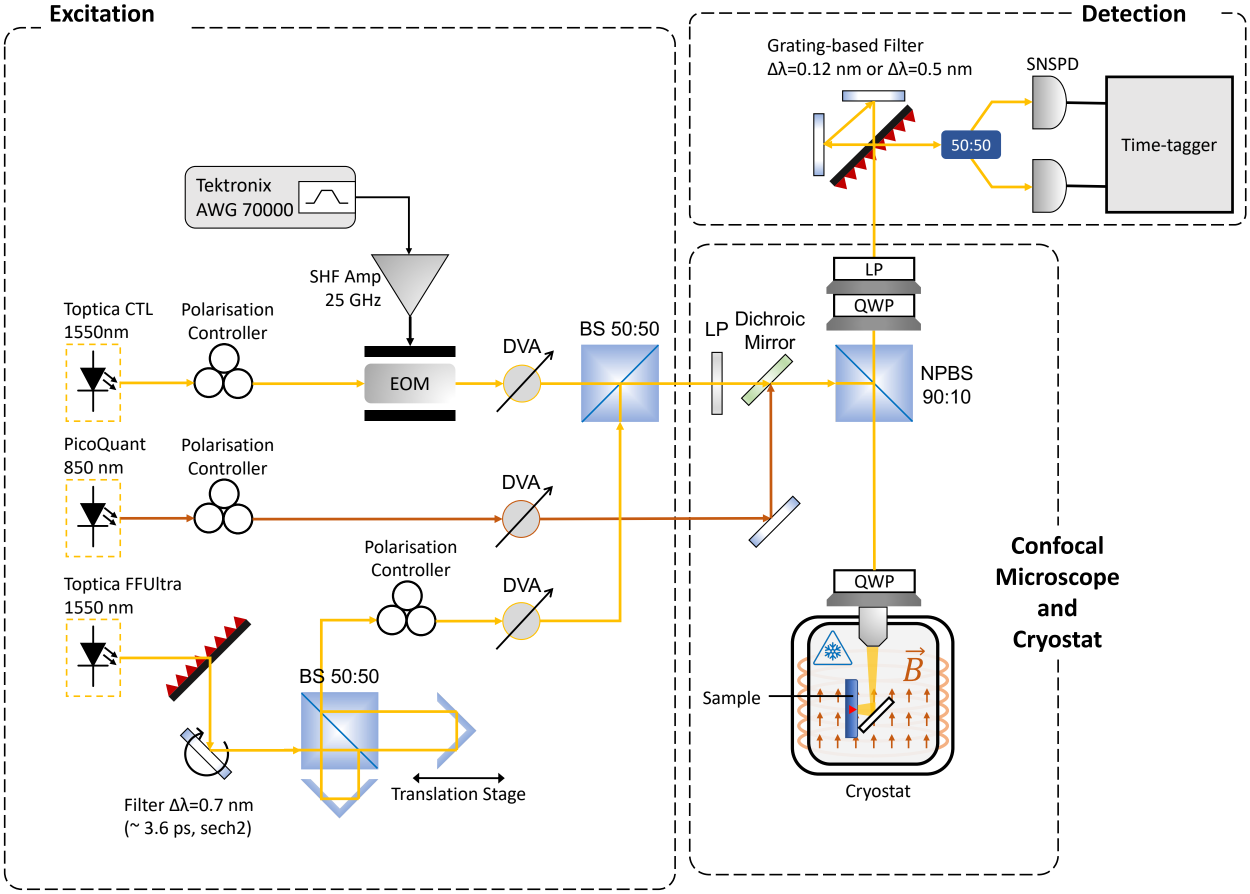

Supplementary Figure 2: **Detailed schematic of the experimental setup.** The setup is used to excite the QD with above-band and/or resonant laser excitation, and couple the scattered light of the quantum dot into a single mode fibre. The output is then detected either with the means of a spectrometer or SNSPDs. EOM, electro-optic modulator; DVA, digital variable attenuator; BS, beam splitter, LP, linear polariser; QWP, quarter-wave plate; NPBS, non-polarising beam splitter; SPCM, single-photon counting module; SNSPD, superconducting nanowire single-photon detector.

Above-band excitation of the QD is performed using a pulsed PicoQuant's LDH 850 nm laser and 40-MHz repetition rate. Resonant excitation is achieved using Toptica's CTL1550 in conjunction with an EOM that is driven by Tektronix's arbitrary wavefunction generator (AWG70000). The pulsed electronic waveforms are generated at 40-MHz repetition rate. Toptica's FemtoFiber Ultra (FFUltra) is used to generate ultra-fast detuned pulses for coherent spin manipulation. The FFUltra pulses are shaped spectrally using a custom-built

free-space grating-based filter. The linewidth of the filtered pulses is ~ 0.7 nm (4 ps) with an average power of 1 mW. For Ramsey interference experiments the pulses go through a Ramsey interferometer where the time delay between the optical pulses is controlled by means of a variable optical delay line. Resonant and non-resonant light is coupled into the excitation arm of the confocal microscope using a dichroic mirror.

The sample is kept in an attoDry1000 closed-cycle magneto-cryostat at a temperature of ~ 4 K. A superconducting magnet surrounding the sample applies a magnetic field up to 9 T perpendicular to the growth direction (Voigt configuration). Attocube piezoelectric stages are used to control the sample's position. The laser pulses are focused onto the sample using an objective lens (NA = 0.68) placed inside the cryostat. The fluorescence light is filtered by a confocal microscope setup to remove the resonant laser contribution via cross-polarisation filtering. Such a cross-polarisation setup can offer excitation resonant light suppression of >4 orders of magnitude. Additionally, an optional quarter-wave plate is added to compensate for any deviations from linear to elliptical polarisation states. These deviations could arise from a birefrigence of the sample, solid immersion lens (SIL), the cryostat window, or the confocal microscope components.

A free-space grating-based filter ($\Delta\lambda \sim 0.5$ nm or $\Delta\lambda \sim 0.12$ nm) is used to spatially separate and couple individual emission lines. The output is either directed to Princeton Instrument's HRS-750-S spectrometer or to IDQ's ID281 SNSPDs with time-tagging electronics. Finally, any remaining background resonant laser contributions are subtracted from the data by recording equivalent data sets without the above band excitation.

To calculate the overall detection efficiency of the device in our setup, we first simulate the first-lens brightness using a 1D-transfer matrix approach [1]. For the NA = 0.68 used here, we estimate a first-lens brightness of 0.15. This, together with further losses in our setup as detailed in Supplementary Table 1, results in a total collection efficiency of about 1%. However, collection efficiency is not the only factor reducing the count rates. From our spectra under non-resonant excitation we expect the X- to be created less than 40% of the time. In addition, excitation efficiency and quantum efficiency will both affect the measured count rate, but are harder to quantify.

Efficiency	Origin
0.15	First-lens brightness from simulations
0.5	Polarisation rejection
0.5	Fibre coupling efficiency
0.4	Grating filter transmission
0.5	Additional losses in detection setup, incl. detector efficiency
0.0075	Total collection efficiency

Supplementary Table 1: Efficiencies of setup components

3. QD STATE SPLITTING AND G-FACTOR

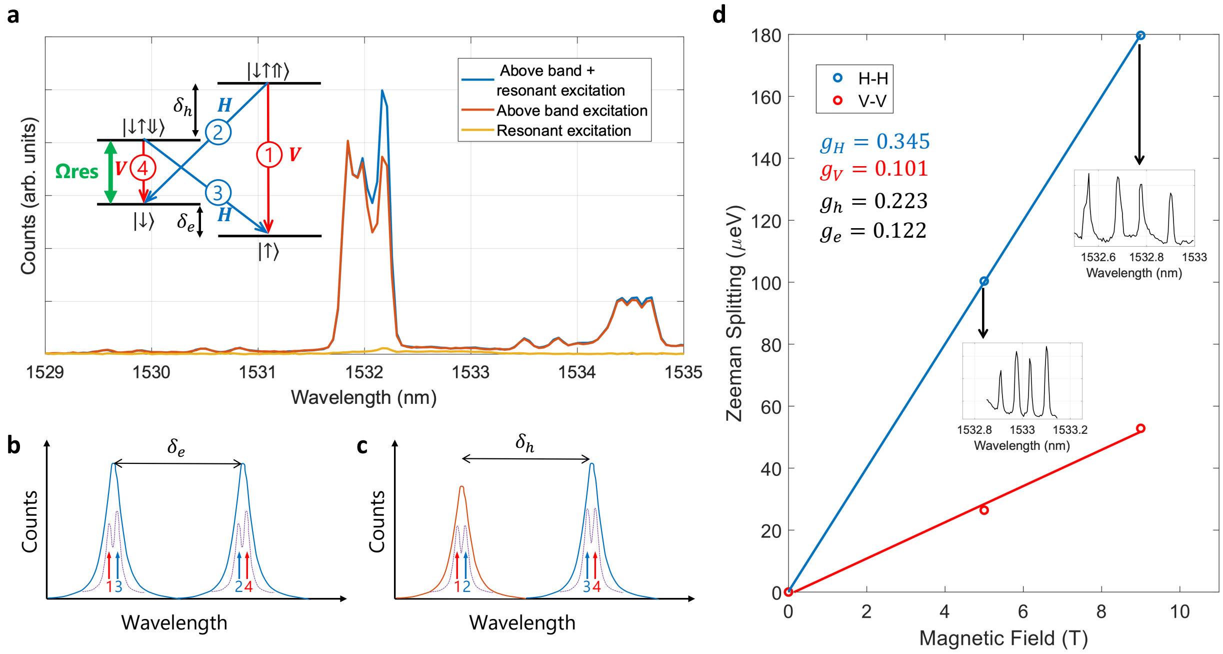

Supplementary Figure 3: **QD state splitting and g-factors under Voigt magnetic fields.** (a) Emission spectra of the QD under above-band, resonant and combined excitation. Inset: Trion level structure with radiative transition selection rules. Transition $|\downarrow\rangle - |\downarrow\uparrow\Downarrow\rangle$ is resonantly driven. (b) and (c) illustrate the two distinct cases where the large energy gap in the emission spectrum corresponds to ground or excited level splitting, respectively. (d) Zeeman splitting between H- and V-polarised transitions as a function of applied magnetic field resulting in excited and ground state g-factors of 0.223 and 0.122, respectively. Insets: Resonant absorption spectra recorded at 5 T and 9 T.

Supplementary Figure 3(a) illustrates the emission spectra under above-band excitation, resonant excitation and combined excitation as recorded by the spectrometer. Under the influence of an external magnetic field, in Voigt geometry, the degeneracy of the eigenstates is lifted and a double Λ−system is formed. The transitions are labelled 1-4 and correspond to the optical selection rules as illustrated in the inset of Supplementary Figure 3(a). Due to the spectrometer's limited resolution, the two higher-energy transitions and lower-energy transitions are lumped into one peak each, with the latter being slightly discernible. The two pairs can be distinguished, around 1532 nm.

The goal is to differentiate between the excited and ground state splittings. The ground state splitting, δ_e, is reflected in the difference between transitions 1-2 and 3-4, the excited state splitting, δ_h, between transitions 1-3 and 2-4. To identify whether δ_h or δ_e correspond to the larger splitting, we resonantly drive the lowest energy transition of the QD and direct the emission to the spectrometer to see which frequencies are emitted. The two possible scenarios are pictorially illustrated in Supplementary Figure 3 (b) and (c). Under above-band + resonant excitation, frequencies 4 and 3 should light up. Thus, if the large splitting is in the ground state, both lumped peaks counts should increase. If the large splitting is in the excited state, only the rightmost lumped peak should increase in counts. From Supplementary Figure 3(a) we can clearly see that the additional intensity due to the resonant excitation is only in one lumped peak. Thus the excited state splitting is larger than the ground state splitting, $\delta_h > \delta_e$.

To determine the g-factor, resonant absorption spectra are recorded as a function of the applied magnetic field. The four distinct transitions are then fitted using Lorentzian functions to determine the energy shifts with respect to zero magnetic field. The energy splitting, Δ_{E_z}, of H and V polarised transitions is plotted in Supplementary Figure 3(d). The g-factors are then given by, $g = \frac{\Delta_{E_z}}{m_j \mu_B \mathbf{B_{ext}}}$, where m_j is the z-component of the total angular momentum, μ_B is the Bohr magneton and $\mathbf{B_{ext}}$ is the applied magnetic field. By performing a linear fit, we extract g-factors $g_H = 0.345$ and $g_V = 0.101$. In turn the excited and ground state g-factors are given by, $g_h = \frac{g_H + g_V}{2} = 0.223$ and $g_e = \frac{g_H - g_V}{2} = 0.122$, respectively.

4. SPIN INITIALISATION THEORETICAL MODEL

To extract the state preparation fidelity, a theoretical rate equation model is developed following Ref. [2]. It is worth noting that the development was an iterative process where at each cycle experimental data and theoretical simulations informed the choice of parameters to be included. The time evolution of all four state populations is modelled with the following rate equations,

$$\frac{d}{dt}n_{|\uparrow\downarrow\Uparrow\rangle}(t) = -2n_{|\uparrow\downarrow\Uparrow\rangle}(t)\Gamma + n_{|\uparrow\downarrow\Downarrow\rangle}(t)\gamma_h - n_{|\uparrow\downarrow\Uparrow\rangle}(t)\gamma_h \underline{\underline{+n_{|\uparrow\rangle}(t)R_p}} \tag{1}$$

$$\frac{d}{dt}n_{|\uparrow\downarrow\Downarrow\rangle}(t) = -2n_{|\uparrow\downarrow\Downarrow\rangle}(t)\Gamma + n_{|\uparrow\downarrow\Uparrow\rangle}(t)\gamma_h - n_{|\uparrow\downarrow\Downarrow\rangle}(t)\gamma_h \underline{\underline{+n_{|\downarrow\rangle}(t)R_{cc}}} \tag{2}$$

$$\frac{d}{dt}n_{|\uparrow\rangle}(t) = n_{|\uparrow\downarrow\Uparrow\rangle}(t)\Gamma + n_{|\uparrow\downarrow\Downarrow\rangle}(t)\Gamma + n_{|\downarrow\rangle}(t)\gamma_e - n_{|\uparrow\rangle}(t)\gamma_e \underline{\underline{-n_{|\uparrow\rangle}(t)R_p}} \tag{3}$$

$$\frac{d}{dt}n_{|\downarrow\rangle}(t) = n_{|\uparrow\downarrow\Uparrow\rangle}(t)\Gamma + n_{|\uparrow\downarrow\Downarrow\rangle}(t)\Gamma + n_{|\uparrow\rangle}(t)\gamma_e - n_{|\downarrow\rangle}(t)\gamma_e \underline{\underline{-n_{|\downarrow\rangle}(t)R_{cc}}} \tag{4}$$

where Γ is the trion spontaneous recombination rate, γ_h and γ_e are the trion-hole and electron spin-flip rates respectively, R_p is the spin pumping rate proportional to the duration of the pump pulse and R_{cc} is the diagonal transitions' cross-coupling rate proportional to R_p. The doubly-underlined terms are only included when the Pump or Probe pulse is ON. The rate equation model is evaluated within four time frames. The 1st (2nd) time frame corresponds to the pump laser being ON (OFF) and similarly the 3rd (4th) time frame corresponds to the probe pulse being ON (OFF). This produces the pump, free evolution, probe and free evolution windows shown in Figure 2(a) of the main text, after charge initialisation has taken place. In this model the Gaussian probe pulse is approximated by a square wave.

To start with, it is assumed that after the non-resonant pulse the charge is injected into a random trion state such that the initial conditions are: $n_{|\uparrow\downarrow\Uparrow\rangle}(0) = 0.5$, $n_{|\uparrow\downarrow\Downarrow\rangle}(0) = 0.5$, $n_{|\uparrow\rangle}(0) = 0$ and $n_{|\downarrow\rangle}(0) = 0$. The spontaneous emission rate is obtained from the non-resonant pulse's decay, $\Gamma = 1.32$ ns. To fit the experimental data the integrated emission intensity is calculated from the theoretical model. A good fit is observed for $R_p = 7.0$ GHz, $R_{cc} = 0.01\%\ R_p$ and $1/\gamma_e = 150$ ns as illustrated in Figure 2(c) of the main text. It is also found that the γ_h does not significantly influence the $|\uparrow\rangle$ state evolution and its fixed to 1 ns^{-1}.

Finally, using the level populations extracted from the best-fit model, the spin-initialisation

fidelity can be calculated as a function of spin-pumping time. The fidelity is defined in terms of states populations at the end of the protocol's cycle,

$$F_{init} = \frac{n_{|\uparrow\rangle}}{n_{|\downarrow\uparrow\Uparrow\rangle} + n_{|\downarrow\uparrow\Downarrow\rangle} + n_{|\downarrow\rangle} + n_{|\uparrow\rangle}} \tag{5}$$

A spin preparation fidelity of 90 % and 95 % is achieved after only 2.2 ns and 8.8 ns of spin-pumping time,respectively, as indicated in Figure 2(c) of the main text.

5. RAMSEY INTERFEROMETRY - ROTATION PULSE FIDELITY

For coherent control of the spin-qubit we employ detuned circularly polarised ps-pulses and make use of the Larmor precession to achieve rotation by θ and ϕ, respectively. For single spin rotations, a broadband high-intensity laser pulse can induce a Stimulated Raman adiabatic Passage (STIRAP). The effective field experience by the QD can be much larger than the applied magnetic field, resulting in an effective Rabi frequency, Ω_{eff} [3].

$$\Omega_{eff} = \sqrt{\frac{\Delta^2|\Omega_H|^2|\Omega_V|^2}{(4\Delta^2+\Gamma^2)^2} + \delta^2} \approx |\Omega_H||\Omega_V|/2\Delta \tag{6}$$

where Ω_j is the Rabi frequency of the j-polarised transition, δ is the Larmor frequency of the ground state and Δ is the detuning of the rotation pulse. The spin state is probed following a excitation by two θ-pulses and separated by a time-delay $\Delta\tau$ set by the Ramsey interferometer. The pulse sequence is illustrated in Figure 3(a) of the main text. After charge injection and spin initialisation, a $\pi/2$-pulse rotates the spin from the north pole to the equator of the Bloch sphere, where it is allowed to precess around the Voigt magnetic field. The Larmor period is defined by the $|\uparrow\rangle - |\downarrow\rangle$ splitting, δ_e. A second $\pi/2$-pulse delayed by $\Delta\tau$ will rotate the spin to either $|\uparrow\rangle$ or $|\downarrow\rangle$. The evolution of the spin-state trajectory is pictorially shown in Figure 3(b). Ramsey fringes are observed in the integrated counts of the readout pulse. The fringe data are fitted with:

$$y(\tau,\theta) = A(\theta)\exp\left(-\frac{\tau}{T_2^*(\theta)}\right)\cos\left(\frac{2\pi\tau}{\delta_e} + \phi(\theta)\right) + B(\theta) \tag{7}$$

where $A(\theta)$ and $B(\theta)$ are the initial amplitude and offset of the Ramsey fringe, $T_2^*(\theta)$ is the inhomogeneous dephasing time, δ_e the Larmor period and $\phi(\theta)$ is the fringe's phase. Note that for long time delays in our Michelson interferometer, there is an increase in noise and a total collapse of Ramsey oscillations, which does not align with an expected decoherence decay, Figure 3(e). While this collapse might be due to technical limitations in our setup, we suspect that dynamic nuclear spin polarisation significantly affects this, complicating the extraction of the T2* coherence time for long delays. To ensure accuracy and reliability, we have excluded long delay data from the fit.

To calculate the fidelity of the $\pi/2$ pulse we follow a similar technique as in [4]. It is assumed that the Bloch vector begins with length A_0 and that with every $\pi/2$ rotation it shrinks by $D_{\pi/2}$. Thus after two $\pi/2$ rotations the population in $|\downarrow\rangle$ oscillates between

$(A_0+A_0D^2_{\pi/2})/2$ and $(A_0-A_0D^2_{\pi/2})/2$. In turn the measured photon counts oscillate between $S(A_0+A_0D^2_{\pi/2})/2$ and $S(A_0-A_0D^2_{\pi/2})/2$, where S is a scale factor to convert the population into the measured counts. These two expressions are then equated to the maximum and minimum of the first Ramsey fringe fit. For a $\pi/2$ rotation we determine that $SA_0 = 1.15$ and $D_{\pi/2} = 0.86 \pm 0.01$. Thus the $\pi/2$-pulse fidelity is estimated to be $F_{\pi/2} = (1+D_{\pi/2})/2 = 93 \pm 0.7\%$.

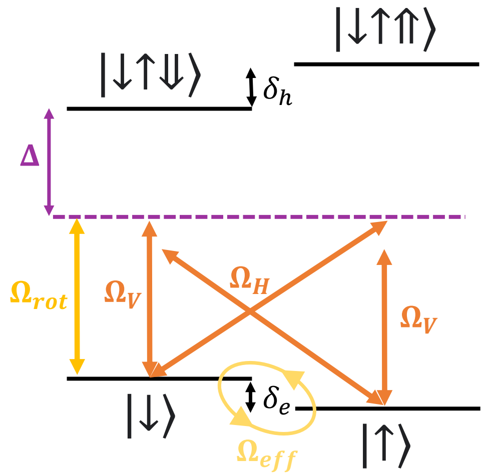

Supplementary Figure 4: Energy level diagram for a negatively charged exciton in a Voigt magnetic field with relevant transitions of the spin rotation scheme.

6. MAGNETIC FIELD OPTIMISATION

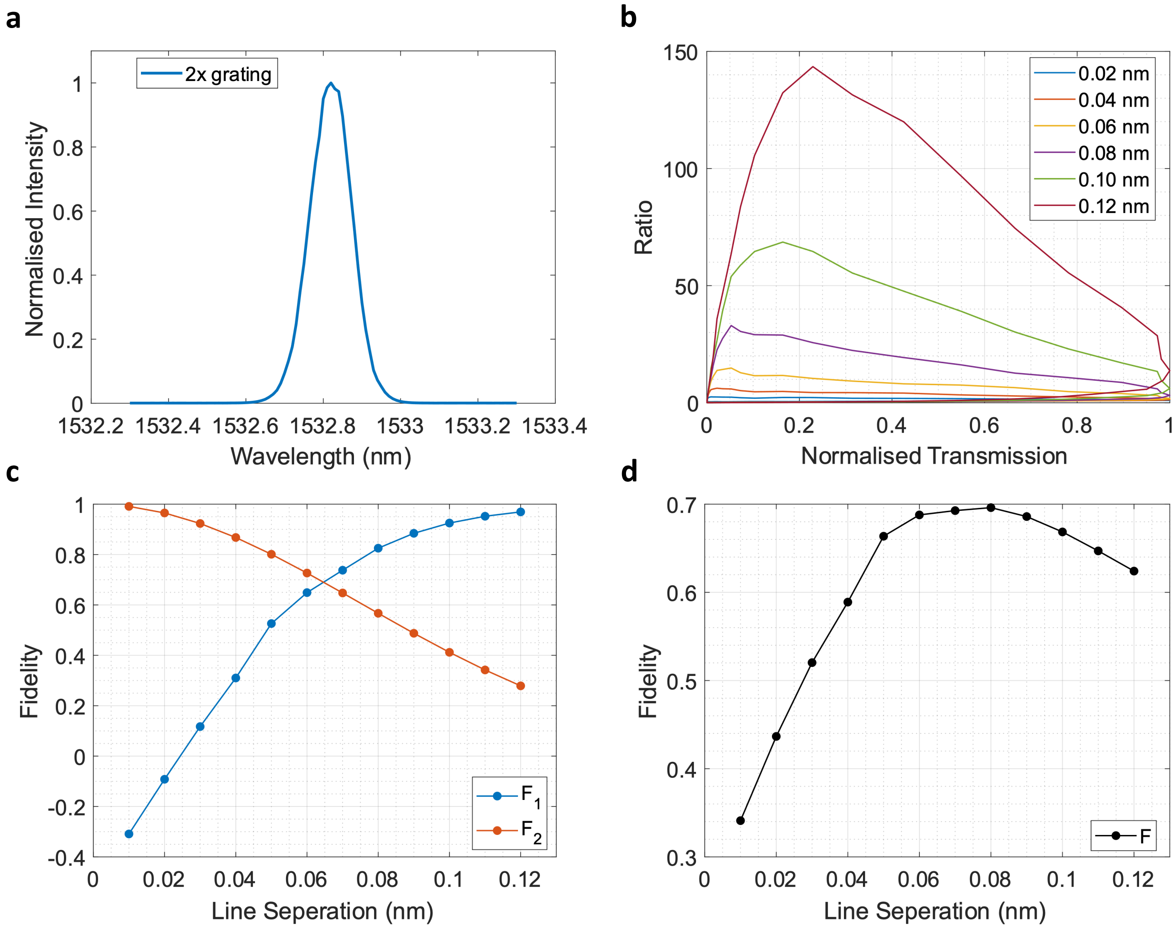

Supplementary Figure 5: **Influence of line splitting on entanglement fidelity.** (a) Measured transmission profile of the double-pass grating filter. (b) Transmission ratio of red vs blue transition, plotted as a function of absolute transmission through the grating (i.e. varying the filter detuning). (c) F_1 and F_2 as a function of line separation. (d) Total expected entanglement fidelity as a function of line separation.

For the spin-photon entanglement experiment, the magnetic field resulting in optimal fidelity given our experimental constraints needs to be determined. For measurements in the computational basis (Figure 4 in the main text), a large splitting is advantageous as it makes filtering the red and blue transitions easier. We use a diffraction grating, which we double-pass, for frequency filtering, and the measured resulting transmission profile is given in Supplementary Figure 5(a). The linewidth of this filter setup is ~ 0.12 nm. The

achievable ratio between red and blue photon transmission depends on the line separation, as well as on the detuning of the filter to take advantage of the steep slopes of the profile. Supplementary Figure 5(b) shows the achievable ratio as a function of transmission of the stronger line through the grating. For efficiency in the experiment, we set the transmission to 0.6. We can then plot the resulting fidelity in the computational basis, F_1, in Supplementary Figure 5(c). As expected, F_1 increases for larger line splittings.

For measurements in the superposition basis (Figure 4 in the main text), where we record interference fringes between the red and blue photons, the detector timing jitter (40-ps FWHM according to the manufacturer) becomes the crucial experimental constraint. The larger the splitting, the faster the beating pattern between the two frequencies, hence a small splitting is preferable for these measurements. To quantify this, we plot in Supplementary Figure 5(c) the predicted beating amplitude after convolution with a 40-ps Gaussian detector response (i.e. F_2) as a function of the line splittings. As expected, the amplitude drops for larger splittings.

These simple estimates for F_1 and F_2 allow us to determine the optimal splitting for the overall entanglement fidelity, which is shown Supplementary Figure 5(d). A broad maximum can be observed for line separations between 0.06 and 0.08 nm. In our experiments, we therefore pick a magnetic field of 5 T (corresponding to a line splitting of 0.065 nm) to maximise the entanglement fidelity without any deconvolution of the measurement in the superposition basis. We further performed the same measurements at a magnetic field of 9 T, to confirm the expected overall behaviour and show that the measurement in the computational basis is mainly limited by the filter resolution.

7. QUANTUM CORRELATIONS AND ENTANGLEMENT FIDELITY

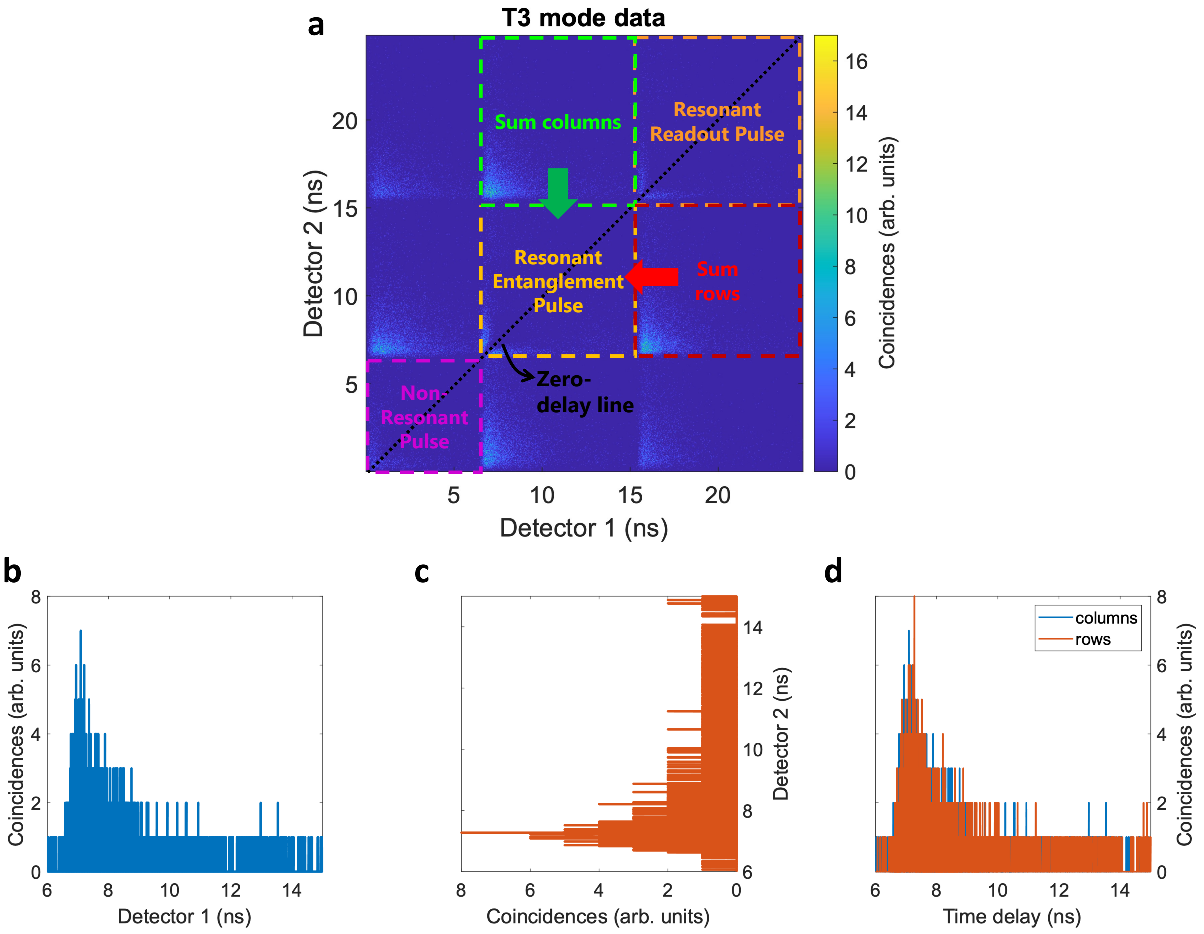

Supplementary Figure 6: **Analysing T3 mode coincidences.** (a) A 2D histogram of photon coincidences, obtained using the HydrHarp in T3 mode, between the two detector channels. The three rectangles along the main diagonal correspond to coincidences amongst the non-resonant and resonant pulses. The green and red rectangles correspond to the region of interest where entanglement correlations are measured. The absence of any coincidences along the main diagonal corresponds to the zero delay line and demonstrates the single photon nature of our source. (b) and (c) show the resulting 1D histograms of arrival times of photons from the entanglement pulse conditioned on the detection of a photon in the readout pulse. (d) Sum of coincidences (b) and (c) in time.

T3 mode

Demonstrating spin-photon correlations requires measuring correlations between the elec-

tron's spin and photon frequency, $|\omega_{red}, \downarrow\rangle$ and $|\omega_{blue}, \uparrow\rangle$.Thus we need to measure coincidences from the entanglement pulse conditioned on the detection of a photon during the subsequent readout pulse. We are only interested in the data where there was an event in the second readout pulse. Due to the dead time of our SNSPDs ($\sim 70ns$) two seprate detection channels will be needed. If a photon emitted from the entanglement pulse is detected, a photon emitted from the readout pulse just a few ns later cannot be detected by the same channel due to the dead time. To get around this problem we split the incoming signal to two detection channels with a 50:50 beamsplitter. Unfortunately, this means that 50% of the coincidences will be lost when photons from the entanglement and readout pulses arrive at the same channel. However, if the photons arrive at different channels a coincidence will be recorded.

To record events with respect with a periodic sync signal we set-up our time-tagger to record events in T3 mode. In brief, T3 mode records the time of an event occurring at each channel w.r.t. to a sync signal and additionally keeps track of the total number of sync signals that have passed. In this way we are able to gain accurate time-domain information for every pulse in our spin-photon entanglement protocol.

Supplementary Figure 6 contains the coincidence data for a quantum correlations. We combine equivalent time-bins from different clock cycles to increase statistics. Note that only cycles along the main diagonal, the zero-delay line, are combined. Also, here we downsample the resulting coincidences from 1 ps to 16 ps to make the figures more readable. Then the coincidences of the entanglement pulse are summed conditioned on the detection of a photon during the subsequent readout pulse. Therefore, there are two regions of interest highlighted by a green/red dashed box in Supplementary Figure 6(a). The coincidences in the green box are summed along the columns and those in the red box along the rows as illustrated in Supplementary Figure 6(b) and Supplementary Figure 6(c), respectively. The histogrammed data are then combined and downsampled to larger time-bins from 1 ps to 8 ps, Supplementary Figure 6(d).

Entanglement fidelity

We calculate the entanglement fidelity, $F = \langle\psi| \rho |\psi\rangle$, using methods developed in [5, 6]. We measure the diagonal elements of the densty matrix in two orthogonal basis and use them to calculate the fidelity in the computational, Equation 8, and superposition basis,

Equation 9.

$$F_1 = \rho_{\omega_{blue}\uparrow,\omega_{blue}\uparrow} + \rho_{\omega_{red}\downarrow,\omega_{red}\downarrow} - 2\sqrt{\rho_{\omega_{red}\uparrow,\omega_{red}\uparrow}\rho_{\omega_{blue}\downarrow,\omega_{blue}\downarrow}} \tag{8}$$

$$F_2 = \rho_{+\rightarrow,+\rightarrow} + \rho_{-\leftarrow,-\leftarrow} - \rho_{+\leftarrow,+\leftarrow} - \rho_{-\rightarrow,-\rightarrow} \tag{9}$$

where $\rho_{ab,ab} = \langle a,b|\,\rho\,|a,b\rangle$ are the diagonal matrix elements and $|\pm\rangle = 1/\sqrt{2}(|\omega_{red}\rangle \mp |\omega_{blue}\rangle)$ and $|\rightarrow\rangle = 1/\sqrt{2}(|\downarrow\rangle - i\,|\uparrow\rangle)$ and $|\leftarrow\rangle = 1/\sqrt{2}(|\downarrow\rangle + i\,|\uparrow\rangle)$.

To extract the entanglement fidelity in the computational basis we make use of the coincidences, $C_{f,s}$, measured in Figure 4 (d,e) of the main text in the time window [1.15 ns, 1.7 ns]. We obtain the density matrix elements $\rho_{fs,fs}$ of Equation 8 by evaluating conditional probabilities $P(f|s)$, as given in Equation 10 and Equation 11, respectively. We summarise the measurements in Supplementary Table 2 and Supplementary Table 3 for 5 T and 9 T, respectively. From the above measurements the fidelities in the computational basis are $F_1^{9T} = 92.87 \pm 1.2\%$ and $F_1^{5T} = 84.13 \pm 1.1\%$.

$$\rho_{fs,fs} = \frac{1}{2}P(f|,s) \tag{10}$$

$$P(f|,s) = \frac{C_{f,s}}{C_{\omega_{red},s} + C_{\omega_{blue},s}} \tag{11}$$

	5 T	
	$f = \omega_{red}$	$f = \omega_{blue}$
$s = \uparrow$	$P(\omega_{red}\|\uparrow) = 3.37 \pm 1.24)\%$	$P(\omega_{blue}\|\uparrow) = 96.63 \pm 0.20\%$
$s = \downarrow$	$P(\omega_{red}\|\downarrow) = 87.62 \pm 0.39\%$	$P(\omega_{blue}\|\downarrow) = 12.38 \pm 0.70\%$

Supplementary Table 2: Conditional probability values $P(f|s)$ at 5 T.

	$f = \omega_{red}$ (9 T)	$f = \omega_{blue}$ (9 T)
$s =\uparrow$	$P(\omega_{red}\vert\uparrow) = 1.10 \pm 1.89\%$	$P(\omega_{blue}\vert\uparrow) = 98.90 \pm 0.12\%$
$s =\downarrow$	$P(\omega_{red}\vert\downarrow) = 94.01 \pm 0.30\%$	$P(\omega_{blue}\vert\downarrow) = 6.00 \pm 0.83\%$

Supplementary Table 3: Conditional probability values $P(f|s)$ at 9 T.

In the rotated basis the application of a $\pi/2$ pulse at time t_{rot} will transform $|\downarrow\rangle \leftrightarrow 1/\sqrt{2}(|\downarrow\rangle + i\,|\uparrow\rangle)$ and $|\uparrow\rangle \leftrightarrow 1/\sqrt{2}(|\downarrow\rangle - i\,|\uparrow\rangle)$. Equation 1 of the main text can now be written as:

$$\frac{1}{2}\left[\left(|\omega_{red},\downarrow\rangle\, e^{-i\omega_z(t_{rot}-t_{phot})} - i\,|\omega_{blue},\uparrow\rangle\right) + i\,|\uparrow\rangle\left(|\omega_{red},\downarrow\rangle\, e^{-i\omega_z(t_{rot}-t_{phot})} - i\,|\omega_{blue},\uparrow\rangle\right)\right] \quad (12)$$

Detection of a photon during the readout pulse will project the electron into $|\downarrow\rangle$. Additionally the small timing jitter of our SSPD's 40 ps compared to $1/\omega_z$ allows us to determine the photon generation time t_{phot} well enough to resolve the time dependence of Equation 12. Therefore the probability of a photon detection is expected to oscillate with $P \sim [0.5 + 0.5\sin(\omega_z(t_{rot} - t_{phot}))]$.

The conditional probabilities in the rotated basis are given by $P_a(+|\rightarrow) = \frac{1}{2}(1 + a_{\rightarrow}\sin(a))$ and $P_a(+|\leftarrow) = \frac{1}{2}(1 - a_{\leftarrow}\sin(a))$ where $a = \omega_z(t_{rot} - t_{phot})$. To obtain $P_a(+|s)$ and $P_a(-|s)$, the coincidences in the rotated basis are normalised using the average number of coincidence events by fitting an exponentially decaying pulse. Then the density matrix elements can be computed using,

$$\rho_{+\rightarrow,+\rightarrow} - \rho_{-\rightarrow,-\rightarrow} = P(\rightarrow)\Big(P_{a=\pi/2}(+|\rightarrow) - P_{a=3\pi/2}(+|\rightarrow)\Big) = \frac{1}{2}a_{\rightarrow} \quad (13)$$

$$\rho_{-\leftarrow,-\leftarrow} - \rho_{+\leftarrow,+\leftarrow} = P(\leftarrow)\Big(P_{a=\pi/2} = (-|\leftarrow) - P_{a=3\pi/2}(-|\leftarrow)\Big) = \frac{1}{2}a_{\leftarrow} \quad (14)$$

Values $a_{\rightarrow}$ and $a_{\leftarrow}$ correspond to the visibility of the oscillations shown in Figure 5 of the main text. To obtain these values we fit the normalised experimental data with $\frac{b_s}{2}(1 \pm a_s\sin(\omega_{z,s}st + \phi)$. We find $a_{\rightarrow} = 0.78(0.06)$ and $a_{\leftarrow} = 0.74{\pm}0.07$ for 5 T and $a_{\rightarrow} = 0.44{\pm}0.09$ and $a_{\leftarrow} = 0.74 \pm 0.12$ for 9 T. The overall measured entanglement fidelity is then,

$$F \geq (F_1 + F_2)/2 \quad (15)$$

SUPPLEMENTARY REFERENCES

[1] Ma, Y., Kremer, P. E. & Gerardot, B. D. Efficient photon extraction froma quantum dot in a broad-band planar cavity antenna. *Journal of Applied Physics* **115**, 023106 (2018).

[2] Dusanowski, Ł. *et al.* Optical charge injection and coherent control of a quantum-dot spin-qubit emitting at telecom wavelengths. *Nature Communications* **13**, 748 (2022).

[3] Clark, S. M., Fu, K.-M. C., Ladd, T. D. & Yamamoto, Y. Quantum computers based on electron spins controlled by ultrafast off-resonant single optical pulses. *Physical review letters* **99**, 040501 (2007).

[4] Press, D., Ladd, T. D., Zhang, B. & Yamamoto, Y. Complete quantum control of a single quantum dot spin using ultrafast optical pulses. *Nature* **456**, 218–221 (2008).

[5] Gao, W. B., Fallahi, P., Togan, E., Miguel-Sanchez, J. & Imamoglu, A. Observation of entanglement between a quantum dot spin and a single photon. *Nature* **491**, 426–430 (2012).

[6] Blinov, B. B., Moehring, D. L., Duan, L.-M. & Monroe, C. Observation of entanglement between a single trapped atom and a single photon. *Nature* **428**, 153–157 (2004).
